# ELOVL6 Genetic Variation Is Related to Insulin Sensitivity: A New Candidate Gene in Energy Metabolism

**DOI:** 10.1371/journal.pone.0021198

**Published:** 2011-06-20

**Authors:** Sonsoles Morcillo, Gracia María Martín-Núñez, Gemma Rojo-Martínez, María Cruz Almaraz, Eva García-Escobar, María Luisa Mansego, Griselda de Marco, Felipe J. Chaves, Federico Soriguer

**Affiliations:** 1 Endocrinology and Nutrition Service, Hospital Carlos Haya, Malaga, Spain; 2 CIBER de Diabetes y Enfermedades Metabólicas Asociadas (CIBERDEM) of the Instituto de Salud Carlos III, Barcelona, Spain; 3 Unidad de Genotipado y Diagnostico Genético, Hospital Clinic Research Foundation Valencia-INCLIVA, Valencia, Spain; University of Cambridge, United Kingdom

## Abstract

**Background:**

The elongase of long chain fatty acids family 6 (ELOVL6) is an enzyme that specifically catalyzes the elongation of saturated and monounsaturated fatty acids with 12, 14 and 16 carbons. ELOVL6 is expressed in lipogenic tissues and it is regulated by sterol regulatory element binding protein 1 (SREBP-1).

**Objective:**

We investigated whether ELOVL6 genetic variation is associated with insulin sensitivity in a population from southern Spain.

**Design:**

We undertook a prospective, population-based study collecting phenotypic, metabolic, nutritional and genetic information. Measurements were made of weight and height and the body mass index (BMI) was calculated. Insulin resistance was measured by homeostasis model assessment. The type of dietary fat was assessed from samples of cooking oil taken from the participants' kitchens and analyzed by gas chromatography. Five SNPs of the ELOVL6 gene were analyzed by SNPlex.

**Results:**

Carriers of the minor alleles of the SNPs rs9997926 and rs6824447 had a lower risk of having high HOMA_IR, whereas carriers of the minor allele rs17041272 had a higher risk of being insulin resistant. An interaction was detected between the rs6824447 polymorphism and the intake of oil in relation with insulin resistance, such that carriers of this minor allele who consumed sunflower oil had lower HOMA_IR than those who did not have this allele (P = 0.001).

**Conclusions:**

Genetic variations in the ELOVL6 gene were associated with insulin sensitivity in this population-based study.

## Introduction

The prevalence of obesity, type 2 diabetes mellitus and risk factors associated with the metabolic syndrome (MS) has increased dramatically over the past 30 years [1], [2]. Insulin resistance is associated with obesity and is the main indicator of early stages of type 2 diabetes [3]. Intracellular lipid accumulation in non-adipose tissue, such as liver and skeletal muscle, is one of the most likely causes of dysfunction of these tissues with regards to insulin resistance [4]–[6]. De novo lipogenesis is a key event for energy metabolism and insulin sensitivity in the liver [7]. Proper elongation and desaturation of fatty acids are essential to the maintenance of lipid homeostasis and disruption of these processes may have important consequences [8]. The elongase of long chain fatty acids family 6 (ELOVL6) is an enzyme which specifically catalyzes the elongation of saturated and monounsaturated fatty acids with 12, 14 and 16 carbons [9]. ELOVL6 is expressed in lipogenic tissues and regulated by sterol regulatory element binding protein 1 (SREBP-1).

Matsuzaka et al demonstrated in Elovl6 −/− mice that the loss of Elovl6 function protected from high-fat induced hyperinsulinemia, hyperglycemia and hyperleptinemia, the fundamental signs of obesity and diabetes, despite the development of obesity and hepatosteatosis [10]. This maintenance of whole-body insulin sensitivity in Elovl6 −/− mice is attributed to continuous signalling through the hepatic IRS-2/Akt pathway, an effect suggested to be mediated by the increased ratio of C16:1 n-7 to C16:0 in the liver [11]. Inhibition of this elongase could be a new therapeutic approach for ameliorating insulin resistance and diabetes, even in the presence of obesity.

To date, all studies on the role of Elovl6 have been undertaken in animal models [10], [12] or are in vitro studies [13]. As far as we are aware, no study has yet been done on ELOVL6 genetic variation in a human population and its possible association with insulin resistance or obesity-related disorders.

We analyzed five SNPs of the ELOVL6 gene using a population-based cohort of persons older than 18 years of age (men and women) in southern Spain, and gathered phenotypic, metabolic, nutritional and genetic information. This is the first study to test the hypothesis that ELOVL6 genetic variation is associated with insulin sensitivity.

## Materials and Methods

### The Pizarra Study

The Pizarra Study is a prospective, population-based cohort study among 1051 subjects aged 18–65 years from Pizarra, a town in the province of Malaga (Andalusia, southern Spain). The study was designed to investigate the incidence and determinants of metabolic disorders, such as type 2 diabetes and obesity. The rationale and design have been described previously [14]–[18]. Informed consent was obtained from each participant, and the study was approved by the medical ethics committee of Carlos Haya Regional University Hospital of Malaga. At baseline (1997–1998), all the participants were interviewed and underwent a standardized clinical examination. The same methods were used at the follow-up study (2003–2004).

### Measures

The same methods were used for both the prevalence study and the incidence study. Standardized measurements were made of weight, height and body mass index (BMI). Persons were considered to be obese if their BMI was 30 kg/m^2^.

The serum was stored at −70°C for later analysis. Blood glucose was measured in both studies using the glucose oxidase method (Bayer, Leverkusen, Germany) at fasting and 120 minutes after an OGTT with 75 g of glucose. Insulinemia at baseline and 120 min after an oral glucose load was measured by radioimmunoassay (Coat-a-Count Insulin, DPC, Los Angeles, CA, USA). Insulin resistance was determined with the formula for the homeostasis model assessment (HOMA_IR) [19]: Fasting insulin (pmol/l) × fasting glucose (mmol/l)/22.5.

Enzymatic methods were used to measure total cholesterol, triglycerides and high-density lipoprotein cholesterol in each sample.

For the baseline study, a sample was taken of the cooking oil being used by a random subset of 538 persons. To avoid the oil being swapped for newer oil, the family was unaware of the intention to request a sample of their oil until the time of the visit by the investigator. All the participants authorized the collection of their cooking oil. The fatty acid composition of the cooking oil was analyzed by gas chromatography [16]. Cooking oil samples were classified according to their fatty acid composition. Considering that only olive and sunflower oils are commercialized in Spain for domestic use, three groups of oils were defined: oils with levels of linoleic acid higher than 50% were classified as sunflower oils, oils with less than 25% linoleic acid were classified as olive oil, and oils containing between 25–50% linoleic acid were classified as mixtures.

### DNA extraction and genotyping

Genomic DNA was isolated from peripheral blood using automatic DNA extraction (Maxwell®16 Instrument, Promega) according to the manufacturer's recommended protocols. DNA was quantified using the RediPlate™ 96 PicoGreen®ystem kit from Molecular Probes and diluted to a final concentration of 10 ng for genotyping.

Five SNPs were selected for genotyping based on their frequency in a Caucasian population (MAF>0.05), their known or possible functional effect, and their position and spacing along the ELOVL6 gene. This information was obtained from the HapMap database (available at http://www.hapmap.org).

SNPs were analyzed by SNPlex (genotyping system based on oligo-ligation assay/polymerase chain reaction technology; Applied Biosystems, Foster City, California, USA).

### Statistical analysis

Hardy-Weinberg equilibrium (HWE) of the five ELOVL6 SNPs was tested using a chi-square test. The continuous variables are shown as the mean and standard deviation and the classification variables as proportions. Calculation of the statistical difference between the means of the continuous variables was done by one-way ANOVA and the qualitative variables by the χ2 test. The strength of association between variables was measured by calculating the odds ratio (OR) and 95% confidence intervals by logistic regression. The multivariate logistic regression model was controlled for potential confounders such as age and sex. In all cases the level of rejection of a null hypothesis was α = 0.05 for two tails. The data were analyzed with the gene analysis program R SNPassoc [20] (version 1.5.8) of the R statistical software, version 2.8.1 (Department of Statistics, University of Auckland, Auckland, NZ; http://www.r-project.org/).

Haplotypes were estimated using also the haplo.stats R package. Haplotypes with frequencies lower than 0.05 were grouped as rare. Linkage disequilibrium between polymorphisms was evaluated by Haploview software version 4.1 (http://www.broad.mit.edu/mpg/haploview).

## Results

### General characteristics

Information on the selected SNPs is shown in **Table 1**. Linkage disequilibrium plot for the five SNPs studied is represented in **Figure S1**.

**Table 1:** Characteristics of ELOVL6 SNPs studied.

Name	Consequence	Sequence	HGVS Names	MAF* Pizarra Study
rs3813825	3PRIME_UTR,DOWNSTREAM	CTTCCTCCTG[T/A]TTGACACTTT	NT_016354.18:g.35515815T>A	0.205
rs17041272	3PRIME_UTR,DOWNSTREAM	GGTTAGAAAC[G/C]GAAGTGAGCA	NT_016354.18:g.35518228C>G	0.058
rs4141123	INTRONIC	AGTTAATTCT[A/G]TAAAATGAAA	NM_024090.1:c.374–747G>A	0.397
rs9997926	INTRONIC	AATGAGCTCA [C/T] GGTTTTCTGC	NT_016354.19:g.35524800C>T	0.103
rs6824447	UPSTREAM	ATAATAAGGA[A/G]CCCTGCTTTT	NT_016354.18:g.35668859G>A	0.476

*MAF: minor allele frequency.

The baseline and follow-up characteristics of the study population are presented in **Table 2**. In total, 824 persons completed the follow-up study. The participation index was 78.5%. Allele and genotype distributions of the 5 SNPs of the ELOVL6 gene followed Hardy-Weinberg equilibrium proportions (P>0.01).

**Table 2:** General characteristics of the study population*.

	Baseline study(n = 824)	Follow-up study(n = 824)
Age (years)	40.6±13.4	46.7±13.9
Sex (male/female) (%)	36.4/63.6	36.4/63.6
BMI(weight/height^2^)(Kg/m^2^)	27.6±5.0	28.7±5.2
Baseline glucose (mg/dl)	96.3±23.4	96.2±23.0
Baseline insulin (µU/ml)	10.4±7.7	9.0±7.5
Triglycerides (mg/dl)	107.2±76.1	99.5±67
Total cholesterol (mg/dl)	199.1±43.2	202.7±39.7
HOMA IR	2.7±2.4	2.5±2.4
HOMA_75 (%)†	37.1	39.9
Obesity (%)	29.3	36.3
Intake of olive oil (%)	75.2%	------

*Persons who completed both the baseline and follow-up studies.

† HOMA75: persons who had HOMA IR above the 75th percentile.

Data are means ± SD or proportions (%).

Obesity: BMI ≥30 kg/m^2^.

### Relation of ELOVL6 variants with HOMA_IR

**Table 3** shows the relationship of the ELOVL6 SNPs with the Homeostasis Model Assessment insulin resistance index (HOMA_IR), insulin and glucose levels at 120 minutes at baseline and in the follow-up study, and the incidence of insulin resistance according to genetic variation. The SNPs rs3813825 and rs9997926 showed a significant association with HOMA_IR at baseline and follow-up study, respectively. To evaluate the influence of the polymorphisms on the risk of having high HOMA_IR values, we selected those persons who had HOMA_IR below the 75th percentile and without type 2 diabetes at baseline. Those persons who had the minor allele (T) of rs9997926 polymorphism had a lower risk of developing insulin resistance six years later (OR = 0.5; CI = 0.29–0.9).

**Table 3:** Glucose levels, insulin levels and HOMA75 in baseline and follow up study according to SNP distribution.

	Baseline study			Follow-up study			Incidence of HOMA75‡
	Insu_120	Gluc_120	HOM75% (%)	Insu_120	Gluc 120	HOM75% (%)	(%)
Rs3813825							
TT	50±46 **b**	118±32 **ab**	33.7 **b**	43±41	118±38 **b**	38.5	28.3
AT	47±40 **b**	116±32 **b**	36.9 **b**	38±31	114±32 **b**	39.6	24.4
AA	77±53 **a**	132±45 **a**	60.0 **a**	57±80	139±63 **a**	53.1	33.3
Rs17041272							
CC	51±46	118±33	36.0	42±41	117±37	39.0	25.9*
CG	50±34	120±33	34.9	45±38	123±44	47.1	38.9
GG	53±31	116±56	60.0	41±21	109±17	40.0	
Rs4141123							
AA	56±41	122±38	38.9	48±55	119±42	41.5	26.3
AG	52±45	117±31	38.7	40±37	118±39	39.3	25.6
GG	48±45	118±33	31.1	42±37	117±34	40.1	30.2
Rs9997926							
CC	50±45	118±33	35.6	43±40	119±38	42.1 **a**	29.8*
CT	52±47	117±33	34.0	38±34	117±40	29.9 **b**	17.9
TT	66±30	130±21	60.0	89±103	122±32	40.0 **ab**	
Rs6824447							
AA	52±45	119±34	40.7	42±34	119±35	44.9	32.8
AG	50±42	118±32	32.4	41±41	118±38	36.9	24.9
GG	52±50	118±34	36.4	45±47	118±40	40.4	25.5

‡Persons who had HOMA IR below the 75th percentile and without type 2 diabetes at the baseline study and who had HOMA IR above the 75th percentile six years later.

Insu_120 =  insulin at 120 min after an oral glucose load; Gluc_120 = glucose at 120 min after an oral glucose load.

*P<0.05. Chi_square test.

Values with different letters are significantly different (P<0.05). ANOVA test.

Insulin (µU/ml), glucose (mg/dl).

Subjects homozygous for the rare allele of rs3813825 (A) had higher insulin and glucose values at 120 minutes in both studies (baseline and follow-up). The rs9997926 polymorphism was also significantly associated with insulin values at 120 minutes in the follow-up study. Only the rs3813825 SNP was significantly associated with the baseline levels of insulin at the initial study. None of the five SNPs was associated with baseline insulin and glucose levels (data not shown).

### ELOVL6 variants and risk of insulin resistance

**Figure 1:**
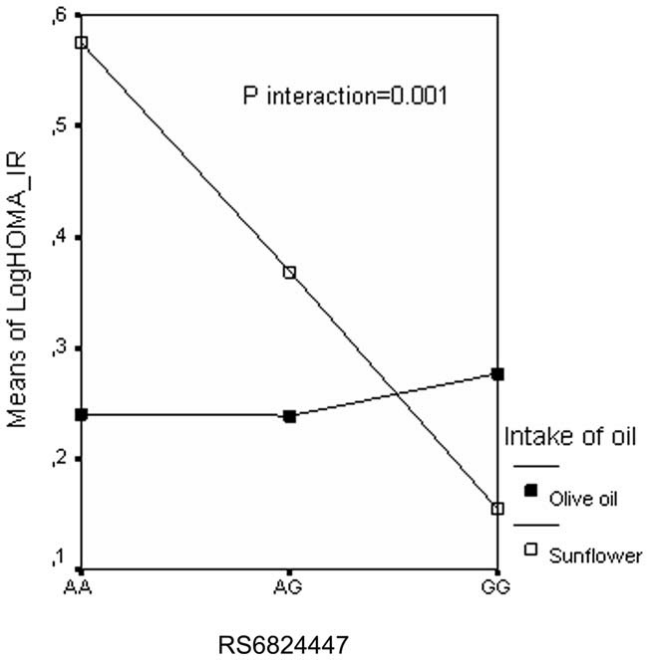
HOMA_IR values by rs6824447 genotypes and intake of oil (olive vs sunflower). Means were adjusted for age, sex and obesity. The P value shown was obtained for the interaction between rs6824447 and intake of oil in a multivariate ANOVA model.

**Table 4** shows the odds ratio (OR) and 95% CI for the risk of developing insulin resistance (HOMA_IR above the 75th percentile) compared with having HOMA_IR values below the 75th percentile for the five ELOVL6 SNPs in the follow-up study. Carriers of the minor allele of rs9997926 (T) had a lower risk of having high insulin resistance values, after adjusting for age, sex and the presence of obesity (OR = 0.54; CI = 0.3−0.8). After adjusting for oil intake (olive vs. sunflower), the minor alleles of the rs17041272 and rs6824447 polymorphisms were significantly associated with the risk of being insulin resistant but with effects in opposite directions. We also found a significant interaction between oil intake and both polymorphisms. **Figure 1** shows the interaction between rs6824447 and oil intake. Subjects homozygous for the rare allele of rs6824447 (GG) and who consumed sunflower oil had lower insulin resistance values than subjects with the AA or AG genotypes. However, no significant difference was found in those persons who consumed olive oil between the various genotypes (P interaction  = 0.001, adjusted for age and sex). A similar finding was observed with the rs17041272 polymorphisms, although this effect only occurred in men (P interaction for men  = 0.04; data not shown).

**Table 4:** Risk of having high values of HOMA_IR (above the 75th percentile) at the **follow-up study** according to ELOVL6 SNPs.

	Model 1*				Model 2†			
	OR* (95% CI)	P cod	P dom	P additive	OR† (95% CI)	P cod	P dom	P additive
Rs3813825								
TT	1.0	NS	NS	NS	1.0	NS	0.06	0.04
AT	1.1(0.8–1.5)				1.4 (0.8–2.3)			
AA	2.3 (098–5.5)				2.3 (0.6–8.5)			
Rs17041272								
CC	1.0	NS	NS	NS	1.0	0.02	0.007	0.01
CG/GG	1.3 (0.8–2.2)				2.6 (1.2–5.4)			
Rs4141123								
AA	1.0	NS	NS	NS	1.0	NS	NS	NS
AG	1.04 (0.7–1.4)				1.5 (0.9–2.5)			
GG	1.1 (0.7–1.8)				1.6 (0.8–3.1)			
Rs9997926								
CC	1.0	0.03	0.01	0.01	1.0	NS	NS	NS
CT	0.5 (0.3–0.8)				0.5 (0.2–1.1)			
TT	0.4 (0.1–1.7)				0.3 (0.04–2.4)			
Rs6824447								
AA	1.0	0.05	0.03	NS	1.0	0.01	0.008	0.006
AG	0.6 (0.4–0.9)				0.5(0.3–0.9)			
GG	0.8 (0.5–1.3)				0.4 (0.2–0.8)			

Logistic regression model.

*Adjusted for age, sex and presence of obesity.

†Adjusted for age, sex, presence of obesity and intake of oil (olive vs sunflower).

P cod: P value for codominant model.

P dom: P value for dominant model.

The codominant model compared the heterozygotes and the homozygotes for the least frequent allele to the homozygotes for the most frequent allele. The dominant model compared a combination of the heterozygous and homozygous genotypes for the least frequent allele to the most frequent homozygous genotypes. The log-additive model is equivalent to calculating the odds ratio for the risk allele.

**Table 5** presents the risk for having high values of insulin resistance after six years in those persons who had HOMA_IR values below the 75th percentile in the baseline study. As with the findings in the follow-up study, carriers of the minor allele of rs17041272 (allele G) had a higher risk for developing insulin resistance compared with non-carriers (or for having HOMA_IR above the 75^th^ percentile). The rs9997926 and rs6824447 SNPs again showed a protective effect against developing insulin resistance, after also adjusting for age, sex, the presence of obesity and oil intake.

**Table 5:** Incidence of HOMA75 after six years in those persons who had HOMA_IR values below the 75th percentile at the baseline study.

	Model 1*			Model 2†		
	OR (95% CI)	P dom	P additive	OR(95% CI)	P dom	P additive
Rs3813825						
TT	1.0	NS	NS	1.0	NS	NS
AT+AA	0.8 (0.5–1.5)			1.2 (0.6–2.3)		
Rs17041272						
CC	1.0	0.07	0.08	1.0	0.005	0.008
CG+GG	1.8 (0.9–3.3)			3.6 (1.2–7.2)		
Rs4141123						
GG	1.0	NS	NS	1.0	NS	NS
AG+AA	0.9 (0.5–1.6)			0.9 (0.5–1.8)		
Rs9997926						
CC	1.0	0.04	0.02	1.0	0.01	0.008
CT+TT	0.5 (0.3–1.0)			0.3 (0.08–0.8)		
Rs6824447						
AA	1.0	NS	NS	1.0	0.04	0.01
AG+GG	0.7 (0.4–1.3)			0.4 (0.2–0.9)		

Logistic regression model.

*Adjusted for age, sex and presence of obesity.

† Adjusted for age, sex, presence of obesity and intake of oil (olive vs sunflower).

P dom: P value for dominant model.

The dominant model compared a combination of the heterozygous and homozygous genotypes for the least frequent allele to the most frequent homozygous genotypes. The log-additive model is equivalent to calculating the odds ratio for the risk allele.

### Haplotypes and insulin resistance

We constructed haplotypes with those polymorphisms that were significantly associated with insulin resistance in our study. Three haplotypes with a frequency of >0.05 were estimated (**Table 6**). The most common haplotype (frequency = 0.459) was used as the reference (Haplo Base). Those haplotypes with a frequency lower than 0.05 were considered as rare haplotypes and they were grouped in one category (hap.rare). Haplotype II was significantly associated with insulin resistance in the follow-up study (OR = 0.54; p = 0.01), with a protective effect. After adjustment for oil intake, haplotype II (TGC) still remained significant, but only just (OR = 0.42; CI = 0.17–1.02; P = 0.05) and haplotype I (CGC) reached statistical significance (OR = 0.69; CI = 0.47–0.99; P = 0.04) **(** **Table 7** **)**.

**Table 6:** Haplotypes estimated in the ELOVL6 gene and observed frequencies in the Pizarra study.

	rs17041272 (C/G)	rs6824447 (A/G)	rs9997926 (C/T)	Frequency
Haplotype 1	C	A	C	0.459
Haplotype 2	C	A	T	0.037
Haplotype 3	C	G	C	0.380
Haplotype 4	C	G	T	0.065
Haplotype 5	G	A	C	0.027
Haplotype 6	G	A	T	0
Haplotype 7	G	G	C	0.03
Haplotype 8	G	G	T	0

**Table 7:** Association analysis between haplotypes and HOMA_IR.

	rs9997926	rs6824447	rs17041272	Freq.	OR (95% CI)	P value
**Unadjusted model**						
Haplo Base	C	A	C	0.458	1.00 (Reference haplotype)	
Hap I	C	G	C	0.379	0.91(0.73–1.2)	NS
Hap II	T	G	C	0.065	0.54(0.32–0.86)	0.01
Hap rare	*	*	*	0.096	0.95(0.66–1.37)	NS
**Adjusted model** ‡						
Haplo Base	C	A	C	0.468	1.00(Reference haplotype)	
Hap I	C	G	C	0.381	0.69(0.47–0.99)	0.04
Hap II	T	G	C	0.053	0.42(0.17–1.02)	0.05
Hap rare	*	*	*	0.097	1.5(0.84–2.67)	0.17
Sunflower vs olive il					2.26(1.34–3.81)	0.002
Age					1.05(1.03–1.07)	<0.001
Sex					0.87(0.54–1.41)	0.58

Logistic regression model. Dependent variable: HOMA_ IR above or below the 75th percentile of the distribution of HOMA IR in the persons with OGTT-N (0 =  HOMA IR ≤75th percentile; 1 =  HOMA IR>75th percentile).

‡ Model adjusted for age, sex and oil intake.

## Discussion

This study shows, for the first time, that genetic variations in the ELOVL6 gene are related with insulin sensitivity in a population from southern Spain. The SNPs rs9997926 and rs6824447 appear to improve insulin sensitivity, noting in our study population that those persons who had the minor alleles of these SNPs had a lower risk of having high HOMA_IR values, whereas those persons who had the minor allele of rs17041272 had a greater risk of being insulin resistant. This effect was seen in both the cross-sectional study and the prospective study.

This study was undertaken in a population of whom 75% consume olive oil, either as the sole source of vegetable fat (50%), or else mixed with sunflower oil (25%); the remaining 25% just use sunflower oil [21]. In this study we tested the effect of the intake of olive oil on the association between the ELOVL6 gene and insulin sensitivity. The results show that the intake of fat (olive oil vs. sunflower oil) modifies the effect of the polymorphisms on insulin resistance, such that those SNPs that were not significant become so after considering the type of oil, thereby implying an interaction between the two factors.

The endogenous synthesis of fatty acids is a crucial step in maintaining lipid homeostasis and an alteration of these pathways can lead to important metabolic changes. More and more pathophysiological syndromes are being related with alterations in lipid homeostasis, and study of the enzymes involved in these processes is taking on increasing importance [8]. Very recent murine studies have shown the importance of the role of Elovl6 in energy metabolism and insulin sensitivity [10]–[12]. Matsuzaka et al showed that deletion of Elovl6 in a mouse model prevents the development of diet-induced insulin resistance, without amelioration of obesity or hepatosteatosis [10]. This deficiency affects not only fatty acid synthesis, but also secondary activation of fatty acid oxidation. Matsuzaka et al suggest that the reduction in insulin resistance in Elovl6 deficient mice could take place via restoration of hepatic insulin sensitivity by phosphorylation of Akt in the liver. Elovl6 deficiency alters hepatic fatty acid composition; changes in fatty acid chain length (decrease in long-chain fatty acids longer than C18) and the ratio of fatty acids (C18:0/C16:0, C16:1/C16:0) could reduce SREBP-1 and PPARα in the liver. Reduction in SREBP-1 leads to decreased fatty acid synthesis via reduction of lipogenic gene expression and increases in IRS-2 levels expression and insulin sensitivity. Reduction in lipogenesis could lead to decreased hepatic diacylglycerol content, which would lead to decreased PKCε activity and increased insulin sensitivity [8], [11].

Several studies have shown that Elovl6 expression is regulated by many dietary, hormonal and developmental factors [22], [23]. This elongase is also regulated by SREBP-1 [12], which in turn is regulated by the PUFA [24]. SREBP-1c plays a crucial role in the dietary regulation of most hepatic lipogenic genes. Matsuzaka et al showed that the mRNA levels of fatty acyl-CoA elongase (FACE) were suppressed in livers from fasted SREBP-1 wild-type mice and markedly activated by refeeding in the wild-type mice, showing nutritional regulation of FACE as a lipogenic enzyme. Similar results were reported by Wang et al [23] in Elovl6 expression levels in the liver of fasted rats that were later refed. Like Matsuzaka, these authors also saw that the PUFA suppressed the expression of lipid enzymes, including Elovl6. FACE mRNA levels are markedly increased in a refed state after fasting in the liver and adipose tissue. This refeeding response is significantly reduced in SREBP-1 deficient mice. Dietary PUFAs caused a profound suppression of this gene expression, which could be restored by SREBP-1c overexpression [12].

The diet in our study population is characterized by a high intake of monounsaturated fatty acids (mainly oleic acid). In this study we found an interaction between the intake of fat (olive oil vs. sunflower oil) and some of the polymorphisms of the ELOVL6 gene in relation to insulin sensitivity. In the case of rs6824447, those subjects with the AA genotype who consumed sunflower oil had higher HOMA_IR values than those who consumed olive oil, whereas these values fell very significantly in the carriers of the minor allele who consumed sunflower oil, there being no variation in the subjects who consumed olive oil. Although the few studies on the nutritional regulation of Elovl6 expression focus on the role of PUFA [12], [25], the effect noted in our study via the MUFA could indirectly reflect the reduction in the levels of PUFA, which in the end would result in a change in the proportion of certain fatty acids, thus affecting the maintenance of lipid homeostasis.

This is the first study to examine the association between genetic variation of the ELOVL6 gene and insulin sensitivity in humans, measured by HOMA as well as hyperinsulinemia at 120 minutes after the OGTT. The causes of postprandial hyperglycemia are influenced by many factors which include a rapid flux of glucose from the gut, impaired insulin release, endogenous glucose production by the liver and peripheral insulin resistance [26], [27]. Our results are in line with those found in animal models and in vitro studies, in which less enzyme activity is associated with better insulin sensitivity. In addition, this study also shows the effect that diet (type of oil consumed) can have on gene function, as was shown in the studies of Matsuzaka and Wang [12], [22], [23].

The strength of this study lies in its prospective nature, with results that were consistent both cross-sectionally and prospectively. These results show that genetic variations in the ELOVL6 gene influence insulin sensitivity in humans. Nevertheless, an important limitation of the study concerns the choice of the SNPs, as these are in linkage disequilibrium with others that could, in fact, account for our findings. Thus, a more profound study of the gene is required in order to detect possible functional variants. The lack of information about the functionality of the genetic variants impedes conclusions from our study as to whether the minor alleles of the SNPs studied are associated with more or less enzyme activity. Nonetheless, from the information currently available and based on our own findings, we can speculate that the SNPs rs9997926 and rs6824447 are related with some functional variant that reduces Elovl6 activity whereas rs17041272 could be linked to a functional variant with the opposite effect. Other limitation of the study concerns the sample size, more studies in bigger population would be necessary to confirm these data.

In summary, we found that carriers of the minor alleles rs9997926 and rs6824447 of the ELOVL6 gene have lower insulin resistance than non-carriers and this effect is not independent of the type of oil consumed. This study supports the results of Matsuzaka, suggesting the importance not only of the degree of fatty acid saturation but also of their length on energy metabolism and insulin sensitivity. The experimental results together with those reported here suggest that the ELOVL6 gene could be a future therapeutic target in the treatment of diabetes and related disorders.

CIBER de Diabetes y Enfermedades Metabólicas Asociadas is an ISCIII project.

## Supporting Information

Figure S1 Localization of single nucleotide polymorphisms in the human ELOVL6 gene. Genomic organization and linkage disequilibrium plot for the five SNPs studied.
(TIF)
