# Supplementary figures and images for: ELOVL6 Genetic Variation Is Related to Insulin Sensitivity: A New Candidate Gene in Energy Metabolism
Source: PLoS One. 2011 Jun 20;6(6):e21198. doi: 10.1371/journal.pone.0021198 (PMC3118791; doi:10.1371/journal.pone.0021198)

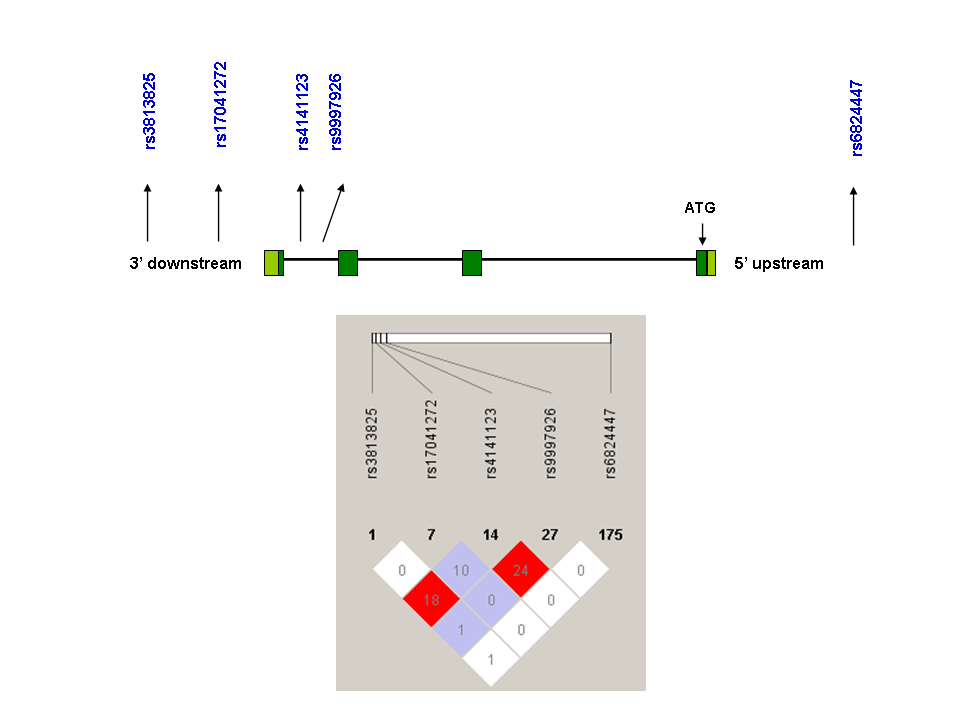

Supplement: Figure S1 — Localization of single nucleotide polymorphisms in the human ELOVL6 gene. Genomic organization and linkage disequilibrium plot for the five SNPs studied. (TIF) [file pone.0021198.s001.tif]
